# Assessment of the Central Effects of Natural Uranium* via* Behavioural Performances and the Cerebrospinal Fluid Metabolome

**DOI:** 10.1155/2016/9740353

**Published:** 2016-05-10

**Authors:** P. Lestaevel, S. Grison, G. Favé, C. Elie, B. Dhieux, J. C. Martin, K. Tack, M. Souidi

**Affiliations:** ^1^Institut de Radioprotection et de Sûreté Nucléaire, Pôle de la RadioProtection de l'Homme, Service de Radiobiologie et d'Epidémiologie, Laboratoire de RadioToxicologie Expérimentale, IRSN, BP 17, 92262 Fontenay-aux-Roses Cedex, France; ^2^Aix Marseille Université (AMU), NORT, 27 boulevard Jean Moulin, 13385 Marseille Cedex 5, France; ^3^Inserm, UMR-S 1062, 27 boulevard Jean Moulin, 13385 Marseille Cedex 5, France; ^4^Inra, UMR-INRA 1260, 27 boulevard Jean Moulin, 13385 Marseille Cedex 5, France

## Abstract

Natural uranium (NU), a component of the earth's crust, is not only a heavy metal but also an alpha particle emitter, with chemical and radiological toxicity. Populations may therefore be chronically exposed to NU through drinking water and food. Since the central nervous system is known to be sensitive to pollutants during its development, we assessed the effects on the behaviour and the cerebrospinal fluid (CSF) metabolome of rats exposed for 9 months from birth to NU* via* lactation and drinking water (1.5, 10, or 40 mg·L^−1^ for male rats and 40 mg·L^−1^ for female rats). Medium-term memory decreased in comparison to controls in male rats exposed to 1.5, 10, or 40 mg·L^−1^ NU. In male rats, spatial working memory and anxiety- and depressive-like behaviour were only altered by exposure to 40 mg·L^−1^ NU and any significant effect was observed on locomotor activity. In female rats exposed to NU, only locomotor activity was significantly increased in comparison with controls. LC-MS metabolomics of CSF discriminated the fingerprints of the male and/or female NU-exposed and control groups. This study suggests that exposure to environmental doses of NU from development to adulthood can have an impact on rat brain function.

## 1. Introduction

Natural uranium (NU) is an alpha particle emitter radionuclide of the actinide series and a ubiquitous environmental trace metal found in almost all types of rocks, soils, plants, and water. Its distribution in the earth is heterogeneous, because of geochemical processes. Surface water and especially ground water play a significant role in the migration and redistribution of this nuclide in the environment. Increased NU levels in groundwater are associated with uranium-rich ores and its high solubility under oxidising conditions in soft and bicarbonate-rich waters [1]. Consequently, populations may be exposed to NU in some countries and regions with high NU levels in drinking water [2–4]. The harmful effects on human health of high levels of NU in drinking water are naturally of great interest to the scientific community and the general public.

NU comprises three isotopes: ^238^U, 99.28%; ^235^U, 0.715%; and ^234^U, 5.5 × 10^−3^%. ^235^U is of particular interest due to its ability to sustain nuclear chain reactions. Techniques have been developed in which uranium ore is chemically enriched, thereby increasing the concentration of ^235^U to 2–4% [5]. The different stages of nuclear fuel cycle lead to the production of enriched uranium (EU) and a by-product with a lower proportion of ^235^U, called depleted uranium (DU). The radiological hazard is more important into the following order: EU > NU > DU, but all these uranium have the same chemical toxicity.

Although the central nervous system is a target organ for many toxic heavy metals and is a radiosensitive organ [6–8] few studies have looked for* in vivo* neurological and neurobehavioural effects following internal contamination with uranium. A few studies on nuclear workers or Gulf War veterans have looked at its brain effects [9–11]. Experimental studies show that after exposure, uranium can reach the brain and lead to neurobehavioural effects on locomotor activity, the sleep-wake cycle, memory, and anxiety [12]. Almost all of these data have been recorded with DU and supraenvironmental levels (i.e., ≥40 mg·L^−1^). The toxicity of DU is expected to be mainly chemical rather than radioactive, so the radiological hazards of uranium have been little investigated.

Several* in vivo* studies have shown that uranium can affect the brain, but a still more sensitive approach is necessary to overcome the specific limitations due to low doses. Metabolomics, the comprehensive analysis of a wide range of metabolites, provides a novel tool in the search for new biomarkers of exposure or diagnostic and is an alternative and complementary approach to establish more -omics techniques such as genomics, transcriptomics, or proteomics. Metabolomics provides the ultimate response of a biological system through the analysis of small molecules (<1000 Da) and the characterisation of metabolic phenotypes. Metabolomics has recently been found efficient for identifying a discriminant metabolic signature of chronic low-dose cesium 137 or uranium contamination in urine of rats [13, 14]. Metabolites in biofluids are in dynamic equilibrium with those in cells and tissues. The metabolites in cerebrospinal fluid (CSF) reflect central nervous system metabolism and the balance between blood and CSF. Their analysis can be helpful in identifying markers of neurological disorders [15].

The purpose of the present work was to establish for the first time whether chronic exposure to NU can induce behavioural effects and whether the CSF metabolome is modified. To mimic environmental contamination of drinking water, especially among children, who are known to be a sensitive subgroup in toxicology [16] and radiobiology [17, 18], male rats were exposed from birth to adulthood, that is, over a continuous 9-month period, through lactation and next drinking water containing concentrations of NU known not to be toxic to the kidneys. We used NU concentrations of 1.5, 10, and 40 mg·L^−1^, since the highest concentration of naturally occurring uranium in spring water is 12 mg·L^−1^ [19]. In order to determine the influence of sex, female rats were exposed to the highest NU concentration (40 mg·L^−1^).

## 2. Materials and Methods

### 2.1. Animals and Exposure

Female Sprague-Dawley rats (*n* = 48) were purchased at gestational day 18 (Charles River, France) and were individually housed under standard conditions (21 ± 1°C) with a 12:00 h/12:00 h light/dark cycle (lights on from 08:00 a.m. to 08:00 p.m.). Animals had free access to food and water. The study was conducted in accordance with French legislation concerning the protection of animals used for experimental purposes. All procedures were performed by scientists certified by the French Ministry of Agriculture (license of first author number 92-254).

At the birth of their pups, mothers were subdivided into four groups (*n* = 12 mothers for each group). One group was contaminated using mineral drinking water supplemented with NU (in its uranyl nitrate form; from AREVA) at a concentration of 1.5 mg·L^−1^ (dose about 0.04 mg·day^−1^ per female rat). A second group was contaminated using mineral drinking water supplemented with NU (in its uranyl nitrate form; from AREVA) at a concentration of 10 mg·L^−1^ (dose about 0.25 mg·day^−1^ per female rat). A third group was contaminated with NU in drinking water at a concentration of 40 mg·L^−1^ (dose about 1 mg·day^−1^ per female rat). The specific activity of NU is 2.42 × 10^+4^ Bq·g^−1^. Pups were exposed to NU throughout lactation* via* the mother's milk. Control mothers drank noncontaminated water (fourth group). After weaning on postnatal day 21, male pups were still exposed to NU* via* drinking water (1.5, 10, or 40 mg·L^−1^) until they reached 9 months of age. Female pups were also still exposed to NU* via* drinking water (40 mg·L^−1^) until they reached 9 months of age. One male or female offspring per litter was assigned to behavioural tests (*n* = 12 for each group). Health parameters, that is, body weight, water consumption, and food intake, were measured at the end of NU exposure (at 9 months of age).

### 2.2. Behavioural Analysis

Male or female pups (*n* = 12 for each experimental group) were submitted to behavioural evaluation tests at 9 months of age. None of the tests required food deprivation, reward, or punishment. The same animals underwent all the tests. Six days are necessary to perform all behavioural tests following this order.

On the first and second days, each animal was individually placed in an open field (45 × 45 cm) and was monitored by an automated activity monitoring system (Bioseb, Chaville, France). Lateral and horizontal movements were recorded over a 15 min session, only on the first day.

On the third day when the rats were acclimated to the open field, they were tested in a two-object recognition task. The animal was placed in the open field with two identical objects for 3 min (first session). After a 1-hour delay, the rat was returned to the open field and allowed to explore two objects, one identical to those presented at the first session (familiar object) and the other different (novel object), for an additional 3 min period (second session). The time spent exploring each object was measured during the 2 sessions [20].

Spatial working memory was assessed on the fourth day in a Y-maze with three arms (70 cm long, 50 cm high, 10 cm wide at the bottom, and 20 cm wide at the top) which converged at an equal angle. The apparatus was placed on the floor of the experimental room. Each rat was placed at the centre of the maze and was allowed to move freely through the maze for a 10 min test session. The sequence and number of visited arms were manually recorded. Alternation was defined as entries in the three different arms, consecutively [21].

Anxiety was assessed on day five in an elevated plus maze comprising a wooden cross at a height of 70 cm with two open (10 cm*∗*70 cm) and two closed arms with walls (10 cm*∗*55 cm*∗*70 cm), arranged such that the arms of the same type were opposite to each other and connected by a common open central platform (5 cm*∗*5 cm). At the beginning of the session, the rat was placed at the centre of the maze always facing the same open arm. The animal was then freed to explore the maze for 5 min. Standard spatiotemporal measures were recorded, including the number of entries in the open and closed arms and the cumulative time spent in the different parts of the maze (open and closed arms). An arm entry was recorded if all four of the animal's paws were in the arm [22]. Between the testing of each animal, the maze was cleaned with a 10% ethanol solution.

The forced swimming test, which was the most stressful test, was performed last, on day 6. The rats were individually placed in a glass cylinder (height of 60 cm and diameter of 40 cm), containing enough water such that the hind legs could not reach the bottom of the cylinder but the tail could. The water was maintained at 23–25°C and the rats were left for 10 min. Immobility was measured during the last 5 min of the test (the animal was judged to be immobile when it floated in an upright position and made only minimal movements to keep its head above water) [23].

All the tests were recorded by a video camera and were read by an observer blind to the exposure conditions.

### 2.3. CSF Sampling and Preparation for Metabolomic Analysis

At the end of behavioural tests, each animal was euthanised with isoflurane, placed prone on the stereotaxic instrument and the head of the rat was fixed in a holder. A terminal CSF sample was obtained by direct insertion of an insulin syringe needle (Myjector, 29G 9 1/200)* via* the arachnoid membrane into the cisterna magna. For this purpose a skin incision was made followed by a horizontal incision in the descending part of the trapezius muscle to reveal the arachnoid membrane. A maximum volume of 100 *μ*L was collected per animal. Each sample was transferred into a polypropylene tube, immediately snap frozen in liquid nitrogen, and stored at −80°C for further analysis. Previous experiments have shown that collecting up to 100 *μ*L using this technique and these conditions provides haemoglobin-free CSF samples.

### 2.4. Metabolomics Analysis

Metabolomics analyses have been performed by Criblage Biologique Marseille (CRIBIOM) platform. For the analysis of CSF, protein of 50 *μ*L was removed by methanol precipitation using 200 *μ*L of cold methanol (−20°C) followed by 5 min centrifugation at 14000 rpm. The supernatant was recovered and filtered through 10 kDa filters to remove all proteins. The extracts were evaporated to dryness under a stream of nitrogen at room temperature and redissolved with 25 *μ*L of water/acetonitrile = 90/10 (v/v). To check for data quality, a blank sample (deionised water) and a pool sample (a mixture of all CSF samples) were extracted/diluted and analysed repeatedly along with the sample series [24].

The samples were analysed on a Dionex UltiMate 3000 (Thermo Fisher Scientific, France) coupled to a Q-Exactive Plus mass spectrometer (Thermo Fisher Scientific, France). The LC conditions were autosampler temperature, 4°C; column temperature, 40°C; solvent flow, 0.4 mL/min (solvent A: water, 10 mM ammonium formate, 0.1% formic acid, and solvent B: acetonitrile, 10 mM ammonium formate, 0.1% formic acid); and gradient, 5% B for 1 min, 5–50% B for 2 min, 50–97% B for 6 min, 97% B for 2 min, 97–5% B for 1 min, and 5% B for 4 min (running time, 16 min). The MS conditions were as follows: acquisition mode, positive electrospray ionisation, and full scan 80–1000* m/z*; capillary voltage, 4.5 kV; capillary temperature, 320°C; cone voltage, 55 V; drying gas flow rate, 8 L·min^−1^.

Multivariate statistical analyses were performed using SIMCA-P+ (version 12, Umetrics). Statistical models were validated by ANOVA in the cross-validation mode, where *p* values less than 0.05 were considered significant. The robustness of the models was assessed by calculating the explained variance values (R2Y) and predicted variances (Q2Y) and by the decrease to negative values of the predicted variance after multiple permutations. Principal component analysis and partial least squares discriminant analysis (PLS-DA) were performed on the processed data in log 10[1 + 10*e* ^9^] and scaled in Pareto mode [25]. Data quality and filtering was performed using appropriately tuned XCMS and less stable features removal [14, 25].

To select the most discriminant variables, we found most appropriate to examine the clustering of features with the variable score values as calculated by hierarchical cluster analysis applied to *w∗c* loadings of the PLS-DA model. The most discriminant mass features were tentatively annotated using MZedDB [26] from the chemical formulas generated from the accurately measured masses (accuracy < 5 ppm) generated by the Thermo Xcalibur Qual Browser molecular formula engine. The KEGG compound ID of any hits was recorded, and all recorded IDs were inserted into the KEGG Mapper (http://www.genome.jp/kegg/tool/map_pathway2.html) for tentative pathway identification.

### 2.5. Uranium Concentrations

Samples (cerebral cortex and CSF) were prepared by adding 8 mL of 70% ultrapure nitric acid and 2 mL of hydrogen peroxide. Samples were then mineralised using a 1000 W microwave (Ethos Touch; Milestone Microwave Laboratory Systems; Begamo, Italy) with a 20 min ramp to 180°C, followed by 10 min at 180°C. The uranium content of samples was determined using an inductively coupled plasma mass spectrometer (ICPMS-VGPQ, EXCELL, Thermo Electron Corporation) with bismuth (1 *μ*g·L^−1^) as internal standard. For uranium, ICPMS limit detection was 10^−4^ *μ*g·L^−1^. Two measurements were performed per sample. Values were expressed as nanograms per gram of fresh tissue or nanograms per *μ*L of CSF and presented as mean ± SEM.

### 2.6. Statistical Analyses

In all the experiments, data are expressed as mean ± SEM and were analysed by two-way ANOVA with the main factors of group and dose.* Post hoc* comparisons were made with the Student-Newman-Keuls test. Differences were considered to be significant if *p* < 0.05 or *p* < 0.01.

## 3. Results

### 3.1. Health Parameters

Body weight was not significantly modified in rats exposed to 1.5, 10, or 40 mg·L^−1^ NU compared with the control group (Table 1). Food intake was also not significantly changed in NU-exposed rats in comparison with control rats (Table 1). Daily water consumption did not significantly change in rats exposed to 1.5, 10, or 40 mg·L^−1^ NU, when compared with control rats (Table 1).

### 3.2. Behavioural Tests in Male Rats

#### 3.2.1. Open-Field Activity

**Figure 1:**
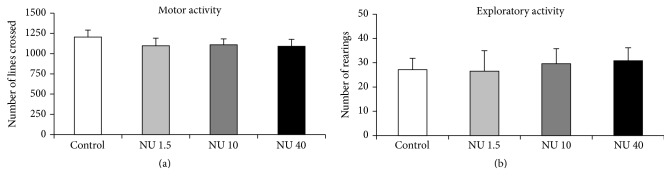
Total activity of male rats exposed to 1.5, 10, or 40 mg·L^−1^ NU from birth for 9 months. (a) shows the number of lines crossed and reflecting locomotor activity. The number of rearings is presented in (b) and reflects exploratory activity. The data are presented as mean ± SEM; *n* = 12 for each group; NU: natural uranium.

The locomotor and exploratory behaviours of rats were assessed by the total number of lines crossed and total number of rearings in the open field over 15 minutes. The results for both parameters are depicted in Figure 1.

In rats exposed to 1.5, 10, or 40 mg·L^−1^ NU, no significant effect on lines crossed or rearing was observed in comparison with controls (Figure 1).

#### 3.2.2. Object Recognition

**Figure 2:**
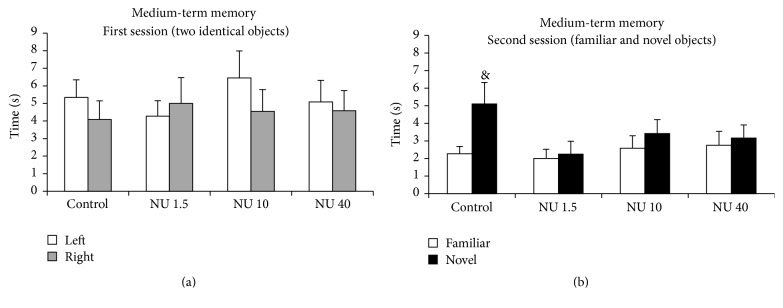
Medium-term memory of male rats exposed to 1.5, 10, or 40 mg·L^−1^ NU from birth for 9 months. (a) shows the time spent exploring one of the two identical objects (left or right objects) during the first session. (b) shows the time spent on the familiar or new objects during the second session. The data are expressed in seconds (s). Results are expressed as mean ± SEM; *n* = 12 for each group; NU: natural uranium; ^&^ *p* < 0.05, significant difference from familiar object.

The medium-term memory of rats was assessed by the time spent exploring novel and familiar objects. The results are shown in Figure 2. During the first session, all groups of animals spent the same overall time exploring the left and right objects (Figure 2(a)). But during the second session, control group rats spent significantly more time exploring the novel object than the familiar object (5.1 ± 1.4* versus *2.3 ± 0.4 s) (Figure 2(b)). This preference for the novel object indicates a memory of the familiar object. Groups exposed to 1.5, 10, or 40 mg·L^−1^ NU did not prefer the novel object to the familiar object (Figure 2(b)). This indicated a loss of medium-term memory in rats exposed to NU without dose effect.

#### 3.2.3. Y-Maze

**Figure 3:**
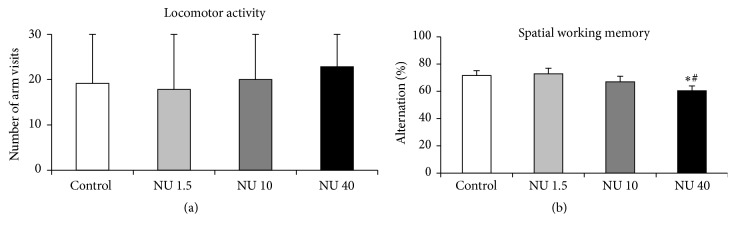
Locomotor activity and spatial working memory of male rats exposed to 1.5, 10, or 40 mg·L^−1^ NU from birth for 9 months. (a) shows the number of arm entries in the Y-maze and reflects locomotor activity. (b) shows the alternation between arms, expressed as a percentage (%), in the Y-maze and reflects spatial working memory. The data are presented as mean ± SEM; *n* = 12 for each group; NU: natural uranium; ^*∗*^ *p* < 0.05, significant difference from control; ^#^ *p* < 0.05, significant difference from 1.5 mg·L^−1^ NU.

The spatial working memory capacities of rats were assessed by spontaneous alternation and number of arm visits, in the Y-maze. The results for both parameters are shown in Figure 3. The percentage alternation was significantly higher than 50%, indicating that spatial memory was present for the four groups.

However, in rats exposed to 40 mg·L^−1^ NU, a significant decrease in the percentage of spontaneous alternation was observed in comparison with the control group (−16%, *p* < 0.05) (Figure 3(b)). This decrease in alternation behaviour was not associated with changes of general locomotor activity, measured as the number of arm visits (Figure 3(a)).

For rats exposed to 1.5 or 10 mg·L^−1^ NU, no significant effect was found on the percentage alternation or on the number of visits to each arm, when compared with controls (Figure 3).

#### 3.2.4. Elevated Plus Maze

**Figure 4:**
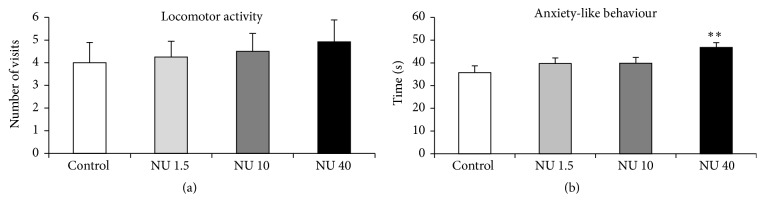
Locomotor activity and anxiety-like behaviour of male rats exposed to 1.5, 10, or 40 mg·L^−1^ NU from birth for 9 months. (a) shows the number of visits to the closed arms of the elevated plus maze and reflects locomotor activity. (b) shows the time spent, expressed in seconds (s), in the closed arms of the elevated plus maze and reflects anxiety level. The data are presented as mean ± SEM; *n* = 12 for each group; NU: natural uranium; ^*∗∗*^ *p* < 0.01, significant difference from control.

The anxiety-like behaviour of rats was assessed by the time spent in the closed arms and the number of closed arm entries in the elevated plus maze. The results for both parameters are shown in Figure 4.

Rats exposed to 40 mg·L^−1^ NU spent significantly more time in the closed arms than did the controls (31%, *p* < 0.01), the rats exposed to 1.5 mg·L^−1^ NU (+18%, *p* < 0.05), and the rats exposed to 10 mg·L^−1^ NU (+18%, *p* < 0.05) (Figure 4(b)). Their number of visits to the closed arms did not differ significantly from that of the other groups (Figure 4(a)).

For rats exposed to 1.5 or 10 mg·L^−1^ NU, no significant difference was observed in the time spent in or the number of visits to the closed arms compared with controls (Figure 4).

#### 3.2.5. Forced Swimming Test

**Figure 5:**
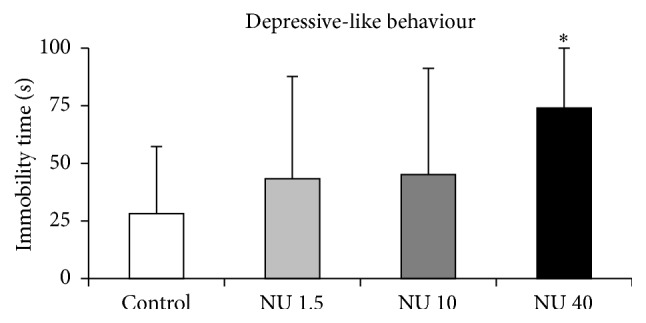
Depressive-like behaviour of male rats exposed to 1.5, 10, or 40 mg·L^−1^ NU from birth for 9 months. Time of immobility is expressed in seconds and reflects the depression level. The data are presented as mean ± SEM; *n* = 12 for each group; NU: natural uranium; ^*∗*^ *p* < 0.05, significant difference from control.

The depressive-like behaviour of rats was assessed by the time they spent immobile during the 5 last minutes of the test. The results are shown in Figure 5. The immobility time did not differ in rats exposed to 1.5 or 10 mg·L^−1^ NU in comparison with control rats (Figure 5), but it increased significantly (+163%, *p* < 0.05) when rats were exposed to 40 mg·L^−1^ for 9 months (Figure 5). As this parameter is usually used to evaluate “depressive-like” behaviour, this result indicates that the depressive-like behaviour was not affected by exposure to 1.5 or 10 mg·L^−1^ NU but increased after 9 months of exposure to 40 mg·L^−1^ NU.

### 3.3. Behavioural Tests in Female Rats at 40 mg·L^−1^ NU

**Figure 6:**
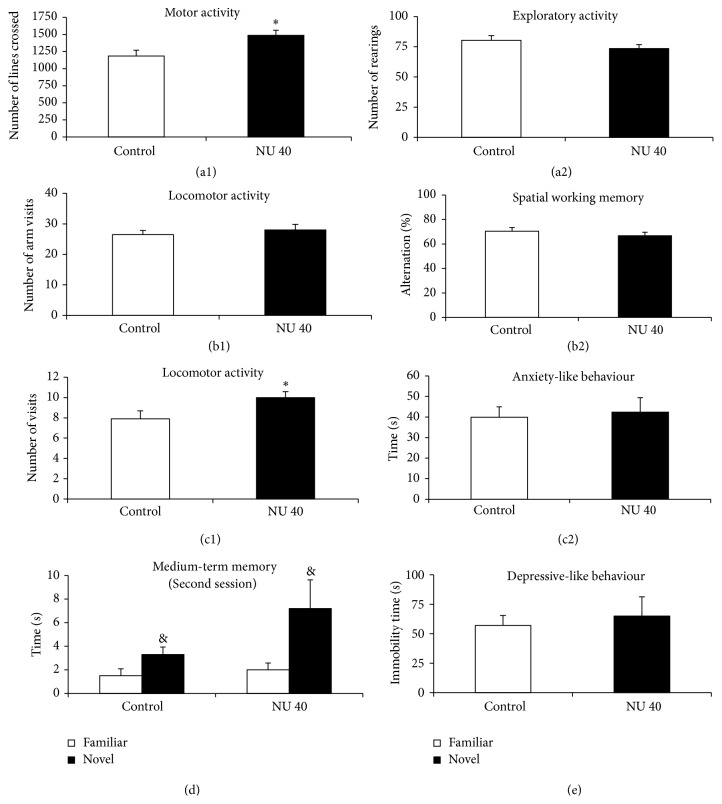
Total activity, spatial working memory, anxiety-like behaviour, medium-term memory, and depressive-like behaviour of female rats exposed to NU at 40 mg·L^−1^ from birth for 9 months. (a1) shows the number of lines crossed, reflecting locomotor activity. The number of rearings is presented in (a2) and reflects exploratory activity. (b1) shows the number of arm entries in the Y-maze and reflects locomotor activity. (b2) shows the alternation between arms, expressed as a percentage (%), in the Y-maze and reflects spatial working memory. (c1) shows the number of visits to the closed arms of the elevated plus maze and reflects locomotor activity. (c2) shows the time spent, expressed in seconds (s), in the closed arms of the elevated plus maze and reflects anxiety level. (d) shows the time spent on the familiar or new objects during the second session expressed in seconds (s) and reflects medium-term memory. (e) shows the time of immobility expressed in seconds (s) and reflects the depression level. Results are expressed as mean ± SEM for control and exposed animals to 40 mg·L^−1^ NU; *n* = 12/group; NU: natural uranium. ^*∗*^ *p* < 0.05, significant difference from control. ^&^ *p* < 0.05, significant difference from familiar object.

The number of lines crossed during the open-field test and the number of closed arm entries in the elevated plus maze increased significantly (+26%, *p* < 0.05 and +27%, *p* < 0.05, resp.) in females exposed to NU compared with controls (Figures 6(a1) and 6(c1)). The number of rearings during the open-field test, spontaneous alternation and the number of arm visits in the Y-maze, the time spent in the closed arms of the elevated plus maze, and the immobility time during the forced swimming test were not significantly modified in female rats exposed to 40 mg·L^−1^ NU compared with control female rats (Figure 6). During the object recognition test, exposed and controls groups spent significantly more time exploring the novel object than the familiar object (Figure 6(d)).

All of these results demonstrated that NU induced behavioural effects linked to the gender. The next step should be the identification of markers of these neurological disorders.

### 3.4. CSF Metabolome

**Figure 7:**
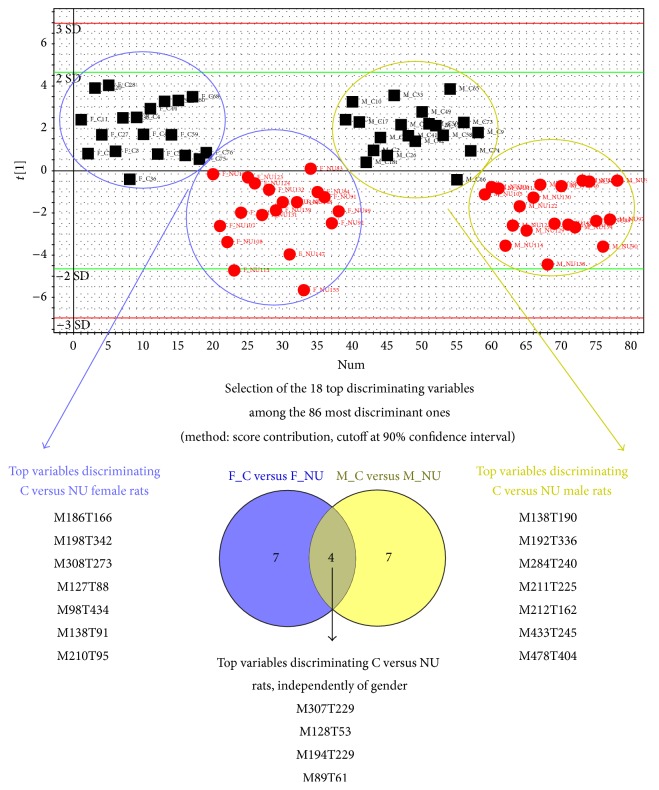
Principal component analysis of the 85 most discriminatory CSF metabolites in male and female rats, control or those exposed to 40 mg·L^−1^ NU from birth for 9 months (PLSDA model built with the 1244 CSF metabolites followed by a hierarchic ascendant classification). C: control; NU: natural uranium.

The variables responsible for the discrimination between control rats and rats exposed to 40 mg·L^−1^ NU are shown in Figure 7. Using principal component analysis, PLS-DA and hierarchical ascendant classification, a model was created with the 86 most discriminating variables from the initial 1244 detected CSF analytical features. This model was built on a single PLS-DA component and it was validated by permutation tests and CV-ANOVA (*p* = 3.91721*e* ^−014^). From this model, we were able to observe and select the best discriminating variables of the 86 significant ones that discriminate control from exposed rats, for male, female, and both animals.

When comparing males to females, from the top 18 control to NU discriminating variables, 7 were found exclusively related to female rats from control rats and 7 others to male rats. Four variables were common for female rats and male rats, corresponding to variables discriminating exposed rats* versus* control rats, regardless of gender (Figure 7). These 4 variables have been putatively identified as N2-succinyl-L-arginine, N4-acetylaminobutanoate, and N-methylsalsolinol, which decreased in NU-exposed rats compared to control rats, and butyric acid, which increased in NU-exposed rats (Table 2).

This metabolomics analysis thus showed that some metabolites differed in the CSF of male* versus* female and the NU-exposed* versus* control groups, whereas others were only specific of NU exposure, irrespective of gender.

### 3.5. Uranium Concentrations

Uranium concentrations in the cerebral cortex of male rats are shown in Table 1. Uranium concentrations in the cortex of male rats exposed to 10 or 40 mg·L^−1^ NU were significantly increased (resp., +110% and +218%) in comparison with control rats (Table 1). No significant difference was observed between rats exposed to 1.5 mg·L^−1^ NU and control rats (Table 1).

The concentration of uranium in CSF increased significantly in male rats exposed to 40 mg·L^−1^ NU compared with control rats (19.7  ±  6.0 ng·L^−1^ *versus *8.1  ±  2.5 ng·L^−1^, *p* < 0.05).

## 4. Discussion

Although there is an undeniable risk of radiological toxicity from orally ingested NU, the hazards of NU have been little investigated, especially after chronic exposure. The primary objective of this experimental study was to obtain new data to shed light on the long-term central effects of NU chronically ingested through drinking water at environmental doses. More specifically, we sought to obtain (i) a phenotypic brain signature associated with NU exposure and (ii) a comparison of the gender related response to NU to reveal any sexual dimorphism associated with exposure. The strength of the present study lies in its combination of a wide range of uranium levels (1.5 to 40 mg·L^−1^), a large panel of behavioural tests (locomotor activity, memory, anxiety, and depression), and the use of a highly relevant, innovative, and sensitive metabolomics approach, which yields a metabolic fingerprint relevant to NU exposure.

Body weight, food intake, and water consumption were evaluated as endpoints of the general toxicity of NU. They were not significantly affected by NU exposure in our experimental conditions, which is in accordance with previous studies in adult rats chronically exposed to DU [27, 28]. We observed no substantial brain weight loss or macroscopic brain tissue damage suggestive of deterioration in health status. These results are not surprising since low doses of NU were used in the present study.

To study the potential adverse effects of uranium on the neurobehaviour of rats chronically exposed to NU, we first investigated total activity. The open-field procedure involves primary motor activity, evaluated by calculating the number of line crossings, and exploratory activity, evaluated by calculating the number of rearings on the hind limbs. In our experimental conditions, motor activity and exploratory activity were not significantly modified in male rats exposed to NU, regardless of the dose. These results are in accordance with a report that there was no significant effect on motor/exploratory activity after 9-month exposure to 40 mg·L^−1^ DU or 4%-EU in adult rats [27]. However, the opposite results, that is, hyperactivity or hypoactivity, in terms of line crossing and rearing or the distance travelled, have also been observed in rats exposed to DU [29–31]. Locomotor activity is closely related to the cholinergic system [32] and exploratory rearing behaviour is evidently related to glutamatergic mechanisms [33]. It is also known that dopamine modulates glutamatergic inputs in the brain [34] and that dopamine-excitatory amino acid interaction is involved in the locomotor behaviour [35]. Verification is therefore needed where uranium-induced impairment of activity is mediated by changes in glutamatergic and dopaminergic neurotransmitters.

We also analysed the effects of NU on emotional behaviour using the forced swimming test, which is generally considered as an animal model of depression [36]. Forced swimming data revealed that male rats that received the highest dose of NU showed increased susceptibility to depressive-like behaviour. The results of the present study suggest also that exposure to 40 mg·L^−1^ NU significantly affects anxiety-like behaviour in the elevated plus maze test, one of the tests most frequently used in behavioural psychopharmacology to assess the potential anxiolytic properties of drugs. This result is consistent with previous data demonstrating a significant effect on anxiety-like behaviour in rats exposed to 4%-EU or DU since birth [27, 37]. Increased depressive-like behaviour is perhaps related to increased anxiety. Monoamine transport systems might play a physiological role in the response to mood disorders induced by uranium. Among them, OCT2, a member of the polyspecific organic cation transporter family, is expressed notably in the limbic system, is implicated in anxiety and depression-related behaviour [38], and could be a target of uranium.

Data obtained with the Y-maze test indicated that exposure of male rats to 40 mg·L^−1^ NU resulted in impairment of spatial working memory, as previously observed in adult rats exposed to 4%-EU for 9 months, but not in adult rats exposed to DU for 9 months [27], suggesting that the percent of ^235^U plays a role. Whatever the dose used in the present experimental study, NU induced a significant decrease in medium-term memory. These results suggest that sensitivity to NU may differ according to the kind of memory studied. The hippocampus for working spatial memory and the hippocampus/entorhinal cortex for medium-term memory may be responsible for this differential sensitivity.

Our behavioural tests showed a strong gender effect, with changes more evidently in males than in females. The single study to date of the involvement of sexual dimorphism in the effects of uranium on behaviour showed increased locomotor activity in male rats, but not females, after ingestion of DU [29]. Brain differences between males and females are a common phenomenon, since sexual differentiation in the brain takes place during a perinatal sensitive window as a result of gonadal steroid hormone-induced developmental organisation [39]. There is considerable evidence for the involvement of sex steroid hormones, such as oestrogen and androgen, in neurotransmitter systems and consequently in possible interactions with cognitive impairment [40]. This difference between males and females heightens the need to investigate this question further.

The brain appears very sensitive to NU. A NOAEL (No-Observed-Adverse-Effect Level) threshold, less than 1.5 mg·L^−1^, may be suggested on the basis of these observed behavioural effects. Dublineau et al. recently demonstrated that the brain is the organ most sensitive to chronic exposure by DU ingestion [41]. These results also revealed that uranium is present in the brain. The next question to consider is how uranium penetrates brain cells. It is becoming evident that uptake transporters are essential in mediating the entry of large numbers of xenobiotics into cells. Some transporters present at the blood-brain barrier, such as organic anion transporting polypeptide 1c1 (Oatp1c1) and monocarboxylate transporter 8 (Mct8), may alter brain development, locomotion, or cognition [42]. This raises the question of their role after NU exposure and thus opens up new perspectives in studying the mechanisms of its toxicity. The exact mechanisms underlying these neurotoxic effects of uranium have not yet been specifically addressed and are probably complex. There are several mechanisms by which uranium may potentially affect the brain. Legrand et al. have demonstrated that some steps of neurogenesis, that is, cell proliferation and cell death, are disturbed during prenatal and postnatal brain development after DU exposure [43]. These effects on neurogenesis could impair synaptic plasticity and might cause cognitive dysfunction in adulthood.

The presence of behavioural impairments suggests that the brain is a target of NU. We used a MS-based metabolomics approach to reveal any metabolic disruption associated to NU exposure that we found highly suitable to reveal low-dose radionuclide contamination [13, 14]. The objective of this preliminary study was to use CSF metabolomics to discriminate between groups of rats exposed or not to NU and to determine whether there is a difference between males and females. We have shown here that some variables specifically discriminated either male and/or female rats exposed to NU from controls at the onset of behavioural deficits, whereas others were only NU specific, irrespective of gender. Among these latter variables that discriminated exposed and nonexposed rats to NU, 4 metabolites were putatively identified as N2-succinyl-L-arginine, N4-acetylaminobutanoate, N-methylsalsolinol, and butyrate. This result demonstrates for the first time that NU has an effect on metabolism of CSF. These metabolites are important for the proper physiological functioning of the brain. For instance, the two first belong to the metabolic pathway of arginine and proline and N-methylsalsolinol is implicated on the balance impairment between dopamine and acetylcholine. These results could be paralleled to studies using metabolomics as a diagnostic marker in neurodegenerative diseases and/or cognitive impairment [44]. Butyric acid which can be found in CSF (database accession number hmdb00039) is also an end-product of ketone bodies metabolism (Kegg pathway map00650), which are compounds used by brain for alternative energy production to glucose. When injected in CSF it has been associated with memory function [45] as well as mood stabilization [46] in rats.

In conclusion, our study demonstrates that the behavioural approach and the application of metabolomics are relevant in the field of low-doses radiation toxicology. Our finding is the first evaluation of the NU-induced health risk in the case of chronic environmental exposure. It suggests that exposure to low-dose NU during development and adulthood can have an impact on behaviour and on the CSF metabolome, highlighting an impact on the brain function and activity in our rat model. The next question is to find out whether and how the changes in the CSF metabolome are related to behavioural changes. The goal now is to continue the identification of these metabolites in order to understand signalling pathways that could explain the behavioural observed effects.

## Figures and Tables

**Table 1:** Body weight, food consumption, water consumption, and cortical uranium concentration of male rats at the end of the experiments.

	Weight (g)	Food consumption (g·day^−1^·rat^−1^)	Water consumption (mL·day^−1^·rat^−1^)	Cortical NU concentration (ng·g^−1^)
Control	650.9 ± 13.5	28.8 ± 0.9	24.9 ± 0.8	0.51 ± 0.06
NU 1.5	639.1 ± 17.2	27.7 ± 0.4	24.8 ± 2.0	0.43 ± 0.04
NU 10	635.5 ± 15.3	28.3 ± 0.6	26.2 ± 2.0	1.07 ± 0.11^*∗∗*^
NU 40	651.5 ± 21.5	28.2 ± 0.8	28.0 ± 2.7	1.62 ± 0.23^*∗∗*^

Results are expressed as mean ± SEM for control and exposed animals to 1.5, 10, and 40 mg·L^−1^ NU; *n* = 12/group; NU: natural uranium; ^*∗∗*^ *p* < 0.01, significant difference from control.

**Table 2:** Putative identification of the variables most responsible for the discrimination between the male and female rats, control, and those exposed to NU at 40 mg·L^−1^ from birth for 9 months.

LC-MS ID	Putative identification	Proposed adduct	Adduct chemical formula	Database ID	Function
M307T229	N2-succinyl-L-arginine	[M + H + CH_3_OH]^1+^	C_11_H_23_N_4_O_6_	C03296	Arginine and proline metabolism

M128T53	N4-acetylaminobutanoate	[M + H − H_2_O]^1+^	C_6_H_10_NO_2_	C02946	Arginine and proline metabolism

M194T229	N-methylsalsolinol	[M + H]^1+^	C_11_H_16_NO_2_	HMDB03892	Endogenous amine found in brain and CSF

M89T61	Butyric acid	[M + H]^1+^	C_4_H_9_O_2_	C00246	Butanoate metabolism, protein digestion, and absorption

NU: natural uranium.
